# CUT homeobox genes: transcriptional regulation of neuronal specification and beyond

**DOI:** 10.3389/fncel.2023.1233830

**Published:** 2023-09-08

**Authors:** Eduardo Leyva-Díaz

**Affiliations:** Instituto de Neurociencias, CSIC-UMH, Sant Joan d'Alacant, Spain

**Keywords:** CUT homeobox genes, *C. elegans*, ONECUT, CUX, SATB, COMPASS, development, disease

## Abstract

CUT homeobox genes represent a captivating gene class fulfilling critical functions in the development and maintenance of multiple cell types across a wide range of organisms. They belong to the larger group of homeobox genes, which encode transcription factors responsible for regulating gene expression patterns during development. CUT homeobox genes exhibit two distinct and conserved DNA binding domains, a homeodomain accompanied by one or more CUT domains. Numerous studies have shown the involvement of CUT homeobox genes in diverse developmental processes such as body axis formation, organogenesis, tissue patterning and neuronal specification. They govern these processes by exerting control over gene expression through their transcriptional regulatory activities, which they accomplish by a combination of classic and unconventional interactions with the DNA. Intriguingly, apart from their roles as transcriptional regulators, they also serve as accessory factors in DNA repair pathways through protein–protein interactions. They are highly conserved across species, highlighting their fundamental importance in developmental biology. Remarkably, evolutionary analysis has revealed that CUT homeobox genes have experienced an extraordinary degree of rearrangements and diversification compared to other classes of homeobox genes, including the emergence of a novel gene family in vertebrates. Investigating the functions and regulatory networks of CUT homeobox genes provides significant understanding into the molecular mechanisms underlying embryonic development and tissue homeostasis. Furthermore, aberrant expression or mutations in CUT homeobox genes have been associated with various human diseases, highlighting their relevance beyond developmental processes. This review will overview the well known roles of CUT homeobox genes in nervous system development, as well as their functions in other tissues across phylogeny.

## 1. Introduction: discovery and classification of CUT homeobox genes

**Figure 1:**
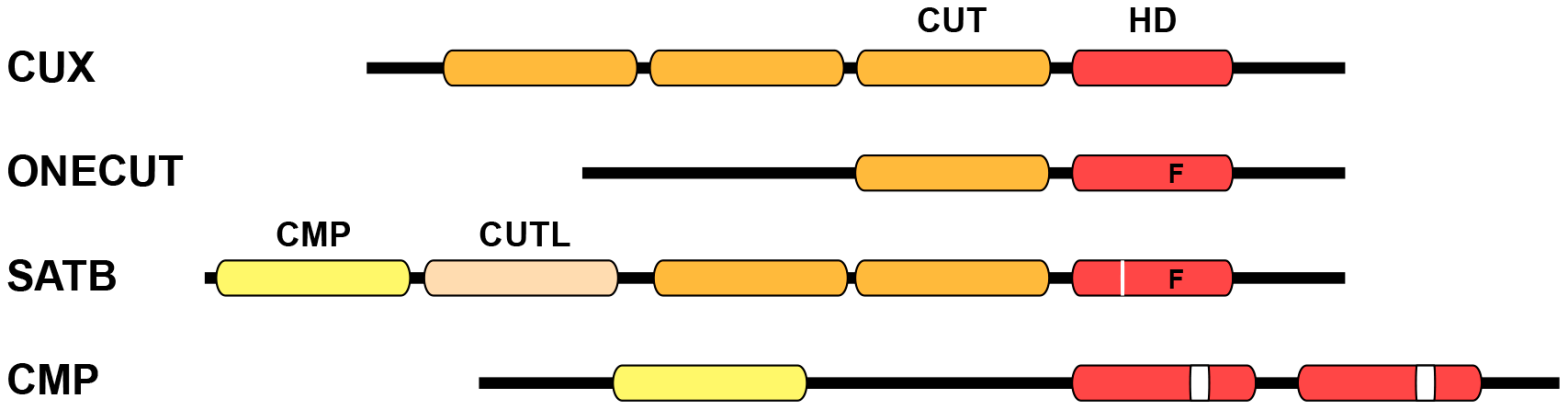
Schematic structure of CUT homeodomain protein families. Particular divergences within the homeodomain are noted below. CUX family proteins are characterized by the presence of three CUT DNA binding domains in addition to a homeodomain. ONECUT family proteins possess a single CUT domain along with a distinct homeodomain that contains a phenylalanine residue at position 48, instead of the canonical tryptophan (letter “F” on scheme). SATB family proteins contain one CMP domain, one CUT-LIKE domain, a couple of CUT domains, and one divergent homeodomain with phenylalanine at position 48 plus an individual residue insertion (white line on scheme). COMPASS proteins are characterized by the presence of the CMP domain, as well as two unconventional homeodomains with a 10 residues insertion (white box on scheme). CUT, CUT domain; HD, homeodomain; CMP, COMPASS domain; CUTL, CUT-LIKE domain.

Homeobox genes encode transcription factors (TFs) that play pivotal roles in development across all multicellular eukaryotes. Extensive evidence has demonstrated their indispensable function throughout various developmental processes, starting from the initial stages in embryonic development to the final stages of differentiation, including neuronal specification in the nematode *Caenorhabditis elegans* (*C. elegans*). Homeobox genes are categorized into several distinct classes attending to the level of sequence similarity within their DNA binding homeodomain (Gehring et al., 1994; Bürglin and Affolter, 2016). The CUT class is a highly conserved class of homeobox genes. This classification derives its name from the pioneering member, *cut*, a homeobox gene intricately associated with the development of external sensory organs in *Drosophila melanogaster* (Blochlinger et al., 1988). The term “cut” originates from a specific wing phenotype characterized by the truncated or abnormal wing development found in different mutants of *Drosophila*. The majority of CUT class homeobox genes contain one or more conserved CUT DNA binding domains, which spans approximately 70 residues. These domains are typically located upstream of the homeodomain. The structure of the CUT domain comprises five primary alpha helixes forming a compact globular domain. The third helix of this domain engages with the major groove of the DNA, establishing sequence-specific interactions (Iyaguchi et al., 2007; Yamasaki et al., 2007). Based on the associated domains, the CUT class has been divided into four distinct families: CUX, ONECUT, SATB, and COMPASS (Figure 1; Table 1; Bürglin and Cassata, 2002; Bürglin and Affolter, 2016).

**Table 1:** CUT homeobox gene complement across phylogeny.

	*CUT homeobox gene class*							
		CUX		ONECUT		SATB		COMPASS
	*#*	Gene names	*#*	Gene names	*#*	Gene names	*#*	Gene names
Worm (*C. elegans*)	1	*ceh-44*	6	*ceh-48, ceh-38, ceh-41, ceh-39, ceh-21, ceh-49*	0		1	*dve-1*
Fly (*D. melanogaster*)	1	*cut*	1	*onecut*	0		1	*dve*
Amphioxus (*B. floridae*)^1^	1	*Bf-Cux*	1	*Bf-Onecut*	0		1	*Bf-Compass*
Ascidian (*C. intestinalis*)	0		1	*Ci-HNF6*	0		0	
Mouse (*M. musculus*)	2	*Cux1, Cux2*	3	*Onecut1, Onecut2, Onecut3*	2	*Satb1, Satb2*	0	
Human (*H. sapiens*)	2	*CUX1, CUX2*	3	*ONECUT1, ONECUT2, ONECUT3*	2	*SATB1, SATB2*	0	

CUT homeobox gene complement in *Caenorhaditis elegans*, *Drosophila melanogaster*, *Branchiostoma floridae*, *Ciona intestinalis*, *Mus musculus*, and *Homo sapiens*.

1 *B. floridae* encodes for an additional CUT class gene (*Bf-Acut*) with a unique domain structure (Takatori and Saiga, 2008).

*Cux* (CUT homeobox) genes encode three CUT domains (also known as Cut repeats) upstream of a homeodomain (Figure 1). The splice variants of Cux genes encode protein isoforms that possess distinct combinations of DNA binding domains (Sansregret and Nepveu, 2008). Biochemical analysis of the human CUX1 was pivotal in providing the first indication that identified the CUT domain as a DNA binding domain (Neufeld et al., 1992). *Onecut* genes encode a unique CUT DNA binding domain and an atypical homeodomain (Figure 1), and probably represent the most ancestral form (Lemaigre et al., 1996; Lannoy et al., 1998). The abundance of CUX and ONECUT genes displays significant variations throughout the animal phylogeny (Table 1; Bürglin and Cassata, 2002; Takatori and Saiga, 2008). For example, vertebrates contain two *Cux* and three *Onecut* genes, whereas the ascidian *Ciona intestinalis* only contains a single *Onecut* gene representing the CUT class homeobox genes (Wada et al., 2003). *Drosophila* contains one *Cux* gene (the founding member *cut*) and one *Onecut* gene (*onecut*). *ceh-44* stands as the only member of the CUX family in *C. elegans*, while the worm contains six different *Onecut* genes which have appeared by diversification and duplication within nematodes (Bürglin and Cassata, 2002).

The SATB (Special AT-rich Binding protein) family of genes encodes two CUT domains and one atypical homeodomain (Figure 1). Furthermore, these genes are characterized by the presence of a COMPASS (CMP) domain at their N-terminus, which is followed by a DNA-binding CUT-LIKE domain (Wang et al., 2014). SATB proteins exhibit binding affinity for AT-rich sections in matrix attachment regions (MARs) and engage in interactions with chromatin modifying proteins and TFs to regulate gene expression (Dickinson et al., 1992; Yasui et al., 2002; Cai et al., 2006). *Satb* genes have only been found in vertebrates, with two family members defined in mouse (*Satb1* and *Satb2*) and other species. The ubiquitin-like structure of the CMP domain has the ability to form tetramers, which indeed allows SATB function, since oligomerization is essential for high affinity DNA binding (Zheng et al., 2017).

Finally, the COMPASS family of homeobox genes stands out as a rare group within the CUT class, as they lack CUT domains. COMPASS genes encode a CMP domain and two divergent homeodomains (containing a 10 amino acid insertion) (Figure 1; Bürglin and Affolter, 2016). The connection to the CUT homeobox genes is established through the presence of the CMP domain, which is exclusive to COMPASS factors and vertebrate SATB proteins. Members of the COMPASS family have been identified in various organisms, including *C. elegans* (*dve-1*), *Drosophila* (*dve*) and the amphioxus *Branchiostoma floridae* (*Bf-Compass*), but not in vertebrates (Bürglin and Cassata, 2002; Takatori and Saiga, 2008).

## 2. Expression patterns of CUT homeobox genes

CUT homeobox gene expression patterns vary across different species but present a consistent and predominant expression in the nervous system. *C. elegans* neurons express six different CUT genes. Among these, the sole *Cux* gene in worms, *ceh-44*, and the *Onecut* gene *ceh-48* show a pan-neuronal expression pattern, while the *Onecut* genes *ceh-38*, *ceh-41, ceh-39* and *ceh-21* are ubiquitously expressed. Nervous system expression is observed for all six genes throughout all developmental stages starting in embryos and maintained in the adult stage. The last *Onecut* gene in the worm genome, *ceh-49*, exhibits expression exclusively during the early stages of embryonic development, prior to neurogenesis (Reilly et al., 2020; Leyva-Díaz and Hobert, 2022). Similarly *Drosophila,* the sea urchin *Strongylocentrotus purpuratus* and the ascidian *Ciona intestinalis* present a unique *onecut* gene broadly expressed throughout the central nervous system (Nguyen et al., 2000; Poustka et al., 2004; Lowe and Stolfi, 2018).

In *Drosophila*, the expression of *cut* within the nervous system is observed in precursors as well as postmitotic cells. However, *cut* expression is not restricted to neurons. During embryonic development, the expression of the *cut* gene is observed in multiple regions, including sensory organs, the central nervous system, the tracheal system, and the Malpighian tubules. Moreover, *cut* expression can be detected in cells located in the prospective wing margin, and its expression patterns are associated with the wing phenotype observed in different *cut* mutants. In the adult, *cut* is expressed in the nervous system, Malpighian tubules, the reproductive system and muscles (Blochlinger et al., 1993).

COMPASS genes also present complex expression patterns: worm *dve-1* is expressed in several regions including neurons, coelomocytes, rectum and intestine. Within the nervous system, *dve-1* is expressed in six distinct neuron classes (Reilly et al., 2020). The fly *dve* is also present in different tissues, including the developing midgut, the wing imaginal disc, and eye photoreceptors (Fuss and Hoch, 1998; Nakagoshi et al., 1998; Kölzer et al., 2003; Johnston et al., 2011).

In vertebrates, CUT homeobox genes exhibit intricate expression patterns both inside and outside the nervous system, with expression in specific regions of the nervous system rather than pan-neuronal (Kropp and Gannon, 2016; Weiss and Nieto, 2019). As overviewed above, vertebrate genomes contain two *Cux* genes, three *Onecut* genes and two *Satb* genes (Table 1). The expression pattern of CUX factors in vertebrates is somewhat reminiscent of that of ONECUT/CUX factors in *C. elegans*: while *Cux1* is found broadly across the embryo and in adult tissues (as ubiquitous worm *Onecut* genes), *Cux2* shows a more specific expression pattern primarily within the nervous system (like *ceh-48* and *ceh-44*) (Iulianella et al., 2003; Nieto et al., 2004). CUX1 exhibits expression in various regions including immune cells, lung, kidney and the genitourinary tract. CUX2 is also found in the genitourinary tract, and in the liver, where it displays a gender specific expression pattern (Iulianella et al., 2003; Conforto et al., 2012, 2015). Within the nervous system, CUX factors are found in key regions such as developing and adult cortical neurons, the hypothalamus, cerebellum and spinal cord (Weiss and Nieto, 2019).

Vertebrate ONECUT factors exhibit expression in various tissues including the liver and pancreas, as well as the nervous system in embryos and adults (Kropp and Gannon, 2016). Their expression and function in the spinal cord has been well documented by several studies (Francius and Clotman, 2010; Stam et al., 2012; Francius et al., 2013; Kabayiza et al., 2017; Harris et al., 2019). In the brain, different studies reported the expression of ONECUT factors in specific regions and neuronal populations including retinal ganglion cells, horizontal cells, dopaminergic neurons, the locus coeruleus or the mesencephalon (Espana and Clotman, 2012a,b; Sapkota et al., 2014). Finally, SATB factors are broadly expressed in multiple tissues. *Satb1* is highly expressed in the thymus and plays a crucial role in T cell development and differentiation (Ahlfors et al., 2010; Hao et al., 2015; Gottimukkala et al., 2016). In addition, *Satb1* expression has also been found in B cells, keratinocytes and testis (Nakagomi et al., 1994; Lena et al., 2012; Ozawa et al., 2022). Within the nervous system, *Satb2* is prominently expressed in the cortex while *Satb1* is detected in various regions within the nervous system, including the cerebral cortex, amygdala, diagonal band, and spinal cord (Dobreva et al., 2006; Huang et al., 2011, 2013). Therefore, considering the expression patterns of the different vertebrate CUT homeobox genes, the majority of neurons in the vertebrate nervous system express a CUT gene at some stage during their development.

CUT homeobox genes play important roles in multiple tissues, with a recurrent involvement in the nervous system, where they determine the specification of postmitotic neurons. The following sections will highlight the diverse functions of CUT homeobox genes both in the nervous system and other tissues.

## 3. CUT homeobox genes define the upper cortical layers

The CUT homeobox genes play critical functions in the development of the upper cortical neurons, including the callosal neurons (Alcamo et al., 2008; Britanova et al., 2008; Weiss and Nieto, 2019). Neurons located in the upper layers of the cortex form intricate networks through cortico-cortical projections. The corpus callosum, the largest fiber tract in the mammalian brain, facilitates communication between the two hemispheres of the cerebral cortex, and is crucial for high-level associative connectivity (Petreanu et al., 2007; Molyneaux et al., 2009; reviewed in Leyva-Díaz and López-Bendito, 2013). Two decades ago, the expression of *Cux1* and *Cux2* was identified as a distinguishing characteristic of the upper cortical neurons and their precursor cells (Nieto et al., 2004; Zimmer et al., 2004). Notably, analysis of ATAC-seq data provided insights into the regulatory interactions of CUX TFs in the cortex, suggesting their repression of key regulators such as *Fezf2*, *Sox5*, and *Nfib*, which are predominantly expressed in the deep layers of the cortex (Gray et al., 2017). In addition, *Cux1* has been found to control the development of upper cortical neurons through the regulation of activity-dependent mechanisms. *Cux1* regulates the Kv1 voltage-dependent potassium channel during development and the postnatal switch to a Kv1-dependent firing mode (Rodríguez-Tornos et al., 2016). This regulatory activity highlights the significance of activity-dependent mechanisms as integral elements of neuronal TF differentiation programs, shaping neocortical circuit assembly.

*Cux2* also has a crucial role in controlling the proliferation of intermediate precursors in the upper layer and promoting their exit from the cell cycle (Cubelos et al., 2008a). This distinct function of *Cux2* is non-redundant with *Cux1*. Vertebrate *Cux* genes are also expressed in the subventricular zone of the medial ganglionic eminence (MGE), where they control the generation of cortical interneurons. Analysis of *Cux1;Cux2* double mutants demonstrated a crucial role for these factors in the specification of Reelin expressing cortical neurons (Cubelos et al., 2008b). Similarly, a recent study found that *Cux2* regulates the number of parvalbumin interneurons generated in the MGE (Magno et al., 2021). Vertebrate CUX proteins are also involved in dendritic arborization, which has been shown *in vitro* (Li et al., 2010), as well as *in vivo*. Analysis of *Cux1* and *Cux2* single knockout mice revealed abnormal dendrites and spines, while the double loss of function resulted in an increased phenotype, highlighting the critical involvement of *Cux* genes in dendritogenesis (Cubelos et al., 2010). Likewise, in *Drosophila*, the *Cux* gene *cut* regulates neuronal progenitors in the external sensory organs (Bodmer et al., 1987; Blochlinger et al., 1988), and also plays an essential role in sensory neurons controlling dendritic arborization (Grueber et al., 2003).

Remarkably, another member of the CUT homeobox gene class was identified as key regulator of upper cortical neurons. At early stages, *Satb2* is expressed in a subset of neurons across the cortical layers, with a notable presence in layers II-IV (Szemes et al., 2006; Alcamo et al., 2008; Britanova et al., 2008). The first analyses of *Satb2* mutant mice showed that cortical neurons fail to cross the corpus callosum and instead project to subcortical regions. In *Satb2* mutants, upper cortical neurons express COUP-TF interacting protein 2 (CTIP2), a crucial TF for layer V corticospinal motor neurons, suggesting that upper layer neurons acquire subcortical projection neuron features in the absence of *Satb2*. However, later works found that the increase on subcortical projections actually originated from deep layer cortical neurons and not from reprogrammed upper layer neurons (Leone et al., 2015; McKenna et al., 2015). Indeed, *Satb2* is expressed at later stages in deep cortical layers, where it is required for proper development of subcortical projection neurons (Leone et al., 2015). *Satb2* directly activates expression of *Fezf2* and *Sox5*, key genes for deep layer cortical neurons development. The mutual regulation between *Satb2* and *Fezf2* explains how *Satb2* leads to subcerebral identity in deep layer neurons, while repressing this very same identity in upper cortical neurons (McKenna et al., 2015; Paolino et al., 2020). Furthermore, during the postnatal stage, *Satb2* is essential in callosal neurons for dendritic and soma adhesion in the cortex, and its absence in adult individuals leads to deficits in long-term memory (Zhang et al., 2012; Huang et al., 2013; Jaitner et al., 2016).

## 4. *Onecut* genes control neuronal specification throughout the nervous system

A function for CUT homeobox genes in neuronal development has been identified in multiple model systems highlighting their critical conserved role. In vertebrates, *Onecut* genes regulate diversification, distribution and maintenance of several specific neuronal populations. In the mouse brain, ONECUT factors are required for the specification of the midbrain dopaminergic nucleus, the locus coeruleus, and the mesencephalic trigeminal nucleus (Chakrabarty et al., 2012; Espana and Clotman, 2012a,b; Sapkota et al., 2014). *Onecut1* interacts with *Lmx1* to facilitate the differentiation of ventral midbrain dopamine neural stem cells into dopamine neurons (Chakrabarty et al., 2012; Yuan et al., 2015). *Onecut2* is essential for the proper neuronal projection and the formation of precise face maps in developing trigeminal neurons (Hodge et al., 2007). In mouse spinal cord, *Onecut* genes have a key function in the generation of specific motor neurons and interneurons. ONECUT factors, in collaboration with a regulatory cascade involving *Neurogenin2*, *Pax6*, and *Nkx6.1*, directly control the expression of *Isl1/2* and *Lmx1a* (Roy et al., 2012; Kim et al., 2015; Kabayiza et al., 2017; Harris et al., 2019). Loss of *Onecut* genes results in a multitude of defects in the central and peripheral nervous systems. These defects include motor neuron atrophy, defects in neuromuscular junctions and in the formation of Renshaw cell interneurons, as well as faulty reorganization of cerebellar Purkinje cells (Audouard et al., 2012; Stam et al., 2012). In addition, ONECUT factors play distinct functions in the development of different regions within the visual system (Emerson et al., 2013; Madelaine et al., 2017). In mice, loss of function experiments have demonstrated that *Onecut1* and *Onecut2* function as regulators of *Prox1* and *Lim1* during the specification and maintenance of multiple cell types in the retina (Wu et al., 2012; Sapkota et al., 2014; Klimova et al., 2015). Understanding the exact roles of Onecut genes in vertebrates has posed challenges due to the existence of multiple gene copies that exhibit overlapping functions. This redundancy has made it difficult to precisely define the specific contributions of each gene. For instance, when both mouse Onecut1 and Onecut2 are simultaneously downregulated, a stronger retinal phenotype is observed compared to the individual mutants (Goetz et al., 2014).

Apart from mice, *Onecut* genes exhibit substantial conservation of function in specifying neuronal cell types across multiple model organisms. In *Drosophila*, *onecut* controls photoreceptor cell differentiation through regulation of the *rhodopsin1* gene (Nguyen et al., 2000). In ascidians, *Onecut* functions in the specification of neurons and photoreceptors (Sasakura and Makabe, 2001). Specifically, the ascidian *Onecut* ortholog plays a crucial role in regulating the retinal homeobox gene *Rx*, which is a key factor involved in eye development in vertebrates (D'Aniello et al., 2011). In *Ciona intestinalis, Neurogenin* was found to activate *Onecut*, which in turn acts in an autoregulatory loop to promote expression of both factors (Pezzotti et al., 2014). The sea urchin ortholog *Strongylocentrotus purpuratus hnf6* (*Sphnf6*) is involved in different functions during embryogenesis, including the formation of the neurogenic ciliated band (Otim et al., 2004). Expression of various *Onecut* orthologs has also been identified in the nervous systems of the sea star *Asterina miniata*, as well as in *Xenopus* and zebrafish (Hong et al., 2002; Otim et al., 2005; Haworth and Latinkic, 2009). Thus, the nervous system stands out as the broadest and most diverse tissue where *Onecut* genes play a fundamental role in regulating cellular specification and differentiation.

## 5. CUT homeobox genes regulate pan-neuronal gene expression

**Figure 2:**
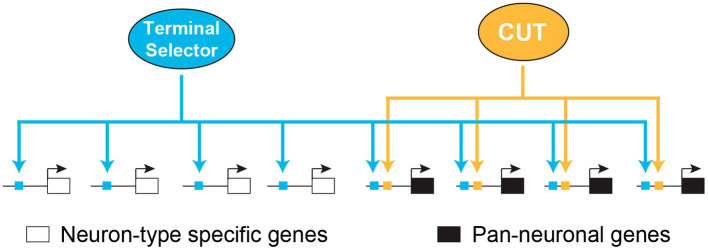
Redundant modular model for pan-neuronal gene regulation. Model illustrating how CUT homeobox genes collaborate with terminal selector transcription factors to regulate pan-neuronal gene expression in *C. elegans*. Adapted from Leyva-Díaz and Hobert (2022). CUT, CUT homeodomain transcription factor.

CUT homeobox genes have been shown to operate with different TF networks to control essential functions in developing and mature cells (Kropp and Gannon, 2016). In a recent study, we have demonstrated that CUT homeodomain TFs play a crucial controlling the expression of pan-neuronal genes in *C. elegans*, encompassing those genes involved in the synaptic vesicle cycle and neuropeptide maturation across all neurons (Leyva-Díaz and Hobert, 2022). CUT factors control pan-neuronal genes by working in collaboration with neuron type specific master regulatory TFs, known as terminal selectors (Hobert, 2011; Hobert and Kratsios, 2019; Figure 2). As mentioned previously, all neurons in *C. elegans* express six distinct CUT genes, starting from embryonic development and maintaining their expression throughout the adult stage. The high sequence similarities in their DNA binding domains predict that these factors recognize the same binding motif. Indeed, available ChIP-seq data for a subset of these CUT factors reveals overlapping patterns of occupancy at pan-neuronal gene promoters. Consistent with this data, CUT binding site deletion disrupts the expression of pan-neuronal genes. Overall nervous system function and pan-neuronal gene expression show minimal defects on single CUT mutants. However, when additional CUT class members are removed, these defects become evident and progressively worsen, highlighting the important function of gene dosage. The rescue of the phenotype in CUT sextuple mutants through overexpressing each individual CUT gene confirms the critical role of gene dosage in CUT gene function. Analysis of neuronal transcriptional profiling in these mutants reveals the requirement of CUT factors for the expression of extensive sets of neuronal genes. These findings provide a significant breakthrough in understanding the specification of neuronal gene expression programs and uncover a highly robust and buffered mechanism that controls essential functional features of all neurons (Leyva-Díaz and Hobert, 2022).

The discovery that human ONECUT1 is capable of rescuing the phenotype of *C. elegans* CUT sextuple mutants implies that vertebrate CUT proteins may also play a role in the regulation of pan-neuronal genes (Leyva-Díaz and Hobert, 2022). This finding suggests a potential conservation of function between vertebrate and invertebrate CUT proteins in the control of gene expression in the nervous system. Interestingly, a study in *Ciona robusta* showed alterations in the expression of synaptic genes when modifying *Onecut* function during photoreceptor differentiation (Vassalli et al., 2021). Another study found that the ONECUT1 binding site is enriched among conserved promoter enhancer elements in vertebrate presynaptic genes (Hadley et al., 2006). Interestingly, ONECUT proteins have the ability to promote neuronal characteristics in a reprogramming approach *in vitro* (van der Raadt et al., 2019). This study highlights the potential of ONECUT factors in driving the acquisition of neuronal features. Indeed, research has suggested that CUT factors play a role in controlling chromatin accessibility and remodeling, indicating their potential as pioneer factors in he establishment of neuronal gene expression programs (Velasco et al., 2017; van der Raadt et al., 2019). These results further support a potential conserved function of CUT factors in vertebrates. Based on genetic loss of function analysis in *C. elegans*, it is predicted that the generation of compound mutants in mice will be required to comprehensively investigate the role of CUT genes in controlling the expression of pan-neuronal genes in vertebrates.

## 6. Multifaceted functions of CUT homeobox genes in non-neuronal cells

Despite their diverse roles in nervous system development observed across species, *Onecut* factors were originally discovered in the context of liver biology. Studies of liver enriched TFs identified a factor activating a gene required for regulating glucose metabolism. Based on its expression pattern and unique DNA binding characteristics, this protein was named Hepatocyte Nuclear Factor 6 (HNF-6), now recognized as ONECUT1 (Lemaigre et al., 1996). The analysis of the HNF-6 protein unveiled the presence of a single domain homologous to the *Drosophila* CUT domain encoded by *cut* and a novel, divergent homeodomain. Based on homology to *Onecut1*, a second and third *Onecut* genes were identified in the liver: *Onecut2* and *Onecut3* (Jacquemin et al., 1999; Vanhorenbeeck et al., 2002). The expression patterns of *Onecut2* and *Onecut3* frequently overlap with *Onecut1* and they share several transcriptional targets. Analyses of *Onecut* mutant mice revealed the essential functions of these factors in the morphogenesis and differentiation of the liver and pancreas (Jacquemin et al., 2003; Margagliotti et al., 2007; Vanhorenbeeck et al., 2007). Interestingly, another CUT homeobox gene, *Cux2*, contributes to sex differences in the liver (Conforto et al., 2012, 2015).

ONECUT factors are expressed both in hepatocytes and in cholangiocytes, the primary cell type constituting the hepatic bile duct. The ONECUT1 DNA binding sequence was described in its specific interaction with *FoxA2*, a regulator of other liver TFs (Samadani and Costa, 1996). Additional studies have highlighted ONECUT1 as a pivotal regulatory factor for numerous genes involved in hepatic development and function, establishing its crucial role in determining the identity of hepatocytes and cholangiocytes. During pancreas specification, *Onecut1* activates several TFs involved in pancreas development including *Pdx1*, *Hnf1β*, *Hnf4α* and *FoxA2* (Briançon et al., 2004; Poll et al., 2006). *Onecut1* plays a crucial role in the differentiation of pancreatic ducts by regulating a network of genes within a specific precursor domain in the developing pancreas (Maestro et al., 2003). The comprehensive review by Kropp and Gannon (Kropp and Gannon, 2016) highlights the similarity between the gene regulatory networks of the developing pancreatic ducts and those of the extrahepatic bile ducts, implying common themes in function. Likewise, they also point to similarities between the retina and the pancreas: in both tissues *Onecut1* acts in parallel with *Ptf1a* to activate gene expression programs for the development of the specific cells (Pierreux et al., 2006; Zhang et al., 2009). In *C. elegans*, apart from its role in neuronal gene regulation, the *Onecut* gene *ceh-39* has been implicated in sexual fate specification (Gladden and Meyer, 2007; Farboud et al., 2013).

*Cux1* exhibits broad, if not ubiquitous, expression, and its functions in non-neuronal cells have been extensively explored through both cell-based assays and studies in mice. Different experiments in cells established the involvement of CUX1 in regulating various cellular processes, including cell proliferation, cell cycle progression, resistance to apoptotic signals, promotion of tetraploidy and concomitant genetic instability, cell invasion and migration, and controlling the response to DNA damage (Michl et al., 2005; Sansregret et al., 2006, 2011; Truscott et al., 2007; Ripka et al., 2010; Vadnais et al., 2012). Studies in mouse models revealed that *Cux1* expression is crucial for hematopoietic system homeostasis (Sinclair et al., 2001). In a different targeted mouse allele, *Cux1^lacZ^*, mice homozygous for *Cux1* mutations on inbred genetic backgrounds exhibited a phenotype characterized by respiratory failure, leading to their premature death shortly after birth (Ellis et al., 2001). A small number of surviving mutant mice exhibited disrupted hair follicle formation and growth retardation. Regarding the *Drosophila Cux* gene, the observed phenotypes in fly mutants reveal that *cut* is involved in cellular specification in multiple tissues, including the Malpighian tubules and the wing (Jack et al., 1991; Liu and Jack, 1992).

SATB proteins have emerged as crucial players in various developmental processes. They contribute to the regulation of specific T-cell developmental stages and influence craniofacial patterning, osteoblast differentiation, erythroid development, embryonic stem cell pluripotency, and hematopoietic stem cell divisions (Alvarez et al., 2000; Nakayama et al., 2005; Wen et al., 2005; Dobreva et al., 2006; Kumar et al., 2007; Savarese et al., 2009; Notani et al., 2010; Will et al., 2013). Additionally, a study has demonstrated the distinctive roles of SATB proteins in the determination of cell lineages through the initial stages of development (Goolam and Zernicka-Goetz, 2017). SATB1 serves as a hub or docking site for numerous chromatin remodeling factors, which repress gene expression (Yasui et al., 2002). This intricate interaction network may provide the mechanistic basis for the diverse developmental functions of SATB1.

COMPASS genes are expressed in various non-neuronal cells, and their functions are only partially understood. *Drosophila dve* is essential for the proper development of the proventriculus and the establishment of the wing disc patterning (Fuss and Hoch, 1998; Nakagoshi et al., 1998; Kölzer et al., 2003). The functions of the worm COMPASS ortholog *dve-1* are still mostly unknown. A number of studies have involved *dve-1* in the regulation of the mitochondrial unfolded protein response (UPR^mt^), a cellular mechanism that is activated in response to stress signals to activate the expression of mitochondrial proteases and chaperones (Merkwirth et al., 2016; Zhou et al., 2022). This mechanism has been investigated in *C. elegans*, revealing that DVE-1 interacts with the histone deacetylase HDA-1 to facilitate a robust UPR^mt^ (Shao et al., 2020).

## 7. Beyond classic transcriptional regulation: SATB factors as architects of chromatin structure

Unlike other homeobox genes, which primarily function as classic TFs, SATB1 was characterized as a unique regulatory protein with a significant role in chromatin modification (Dickinson et al., 1997). SATB1 was identified as a factor implicated in the tissue specific organization and regulation of chromatin, which binds selectively to MARs (Dickinson et al., 1992). The name SATB1, which stands for Special AT-rich sequence Binding protein 1, was derived from early experiments that demonstrated its selective binding to specific AT-rich DNA sequences (Dickinson et al., 1992). The SATB1 homeodomain does not possess the capability to bind directly to DNA. Instead, SATB1 binds to DNA indirectly by interacting with the sugar phosphate backbone structure influenced by a specific DNA sequence context. The two CUT domains and the homeodomain allow SATB1 to identify core unwinding elements with increased affinity and specificity (Yamasaki et al., 2007).

The organization of the mammalian genome involves the formation of intricate higher order configurations that emerge from the multilevel folding of DNA. Within the cell nucleus, chromosomes adopt specific spatial arrangements, forming distinct chromosome territories that are predominantly segregated from one another (Cremer and Cremer, 2001). It has been proposed that within these territories chromatin is folded into chromatin loops, ranging from small loops (approximately 50–200 kb) to giant loops spanning several megabases (Cremer et al., 2006). These giant loops extend beyond their original chromosome territories and intertwine with neighboring territories, facilitating the close spatial proximity of distal genomic loci. This arrangement enables long range interactions between sites, whether they are located on the same chromosome or different chromosomes (Dekker et al., 2002; Spilianakis et al., 2005; Hakim et al., 2012). The formation of loops in chromosomes serves not only to compact the chromatin but also to control gene expression. SATB1 was identified as an architectural chromatin protein controlling gene expression by folding chromatin into loops (de Belle et al., 1998; Cai et al., 2003, 2006; Kumar et al., 2007). Therefore, unlike classic TFs, which control individual genes through specific binding, SATB1 functions by binding to multiple sites where chromatin is anchored to form loop regions. SATB1 acts as a global “genomic organizer” by recruiting various cofactors and chromatin modifying complexes to regulatory elements. This enables SATB1 to coordinate the transcriptional potential of large groups of genes and influence promoter activity by mediating long-range interactions (Galande et al., 2007; Ahlfors et al., 2010; Gong et al., 2011). SATB2 is also involved in the control of chromatin modifications and the regulation of gene expression through its interaction with MARs (Dobreva et al., 2003; Britanova et al., 2005). Similarly, CUX1 was also reported to interact with MARs (Banan et al., 1997; Liu et al., 1999; Kaul-Ghanekar et al., 2004). Numerous studies have provided evidence that when SATB1 is ectopically expressed, it induces significant changes in gene expression and the chromatin landscape of multiple cancer cells, promoting tumor growth and facilitating the metastatic process (Han et al., 2008; see Section 9).

## 8. Beyond classic transcriptional regulation: CUX isoforms and DNA repair

Full-length CUX1 regulation of gene expression also occurs through a non-canonical DNA binding mechanism. Full-length CUX1 protein is abundantly present and exhibits rapid binding to DNA, although the binding is transient in nature (Moon et al., 2000). These characteristics are not typical of classic TF that binds to DNA in a stable manner and recruits cofactors. However, CUX1 can still repress gene expression by competing for binding site occupancy (Mailly et al., 1996). Actually, CUX1 was originally named CDP (CCAAT-displacement protein) (Neufeld et al., 1992). The CCAAT-displacement activity is a mode of transcriptional repression where activators are displaced through competitive binding, a mechanism termed “passive repression” (Luo and Skalnik, 1996; Kim et al., 1997; Stünkel et al., 2000).

A significant finding was the discovery that CUX1 is not present as a single protein species with a uniform mode of DNA binding and control of gene expression. Instead, it can be detected as multiple isoforms with different DNA binding and transcriptional regulation characteristics (Rong Zeng et al., 2000). While a single CUT domain alone is unable to bind to DNA, it can effectively do so in collaboration with another CUT domain or in conjunction with the homeodomain (Moon et al., 2000). Experimental studies using recombinant proteins have provided valuable insights into the DNA binding capabilities of the CUX1 protein. Specifically, these studies have demonstrated that the CUT repeat 1 (CR1) is capable of DNA binding in conjunction with CR2 or in cooperation with the homeodomain. Intriguingly, the CR1CR2 fusion protein exhibits a remarkably distinct DNA binding dynamics. Similar to full-length CUX1, the CR1CR2 fusion protein displays rapid association and dissociation rates with DNA. In contrast, any recombinant protein containing a homeodomain in addition to a CUT domain (excluding CR1) exhibits slower but more stable DNA binding. Indeed the presence of CR1 was shown to determine the DNA binding specificity and kinetics. Proteins harboring the CR1 demonstrate a rapid and transient binding to DNA and display the capacity for CCAAT-displacement activity (Truscott et al., 2004).

Numerous studies have characterized the distinctive characteristics of various CUX1 isoforms, which have been named attending to their apparent molecular weights, including p150, p110, p90, p80 and p75 [reviewed in Sansregret and Nepveu (2008)]. Consistent with this, p200 CUX1 refers to the full length protein. While p75 is generated by alternative splicing, the remaining isoforms are produced via proteolytic processing (Goulet et al., 2002, 2004, 2006; Maitra et al., 2006). The p75, p90, and p110 CUX1 isoforms share common DNA binding and transcriptional characteristics that set them apart from the p200 isoform. Notably, these isoforms exhibit significantly slower DNA binding kinetics in comparison to p200 (Moon et al., 2000; Goulet et al., 2006). Contrary to the full length CUX1 protein, which is primarily involved in transcriptional repression, the p110, p90 and p75 isoforms exhibit a broader range of functions. These shorter isoforms can engage in both transcriptional activation and repression, depending on the specific context of the promoter (Aleksic et al., 2007; Harada et al., 2008; Sansregret and Nepveu, 2008). It is interesting to note here that the p75 isoform, containing the CR3 and the homeodomain, has a very similar structure to that of ONECUT proteins and, if a similar isoform exists in *C. elegans*, it might explain the redundant function of the worm CUX protein CEH-44 with the multiple worm ONECUT factors (Leyva-Díaz and Hobert, 2022). However, it is crucial to note that these isoforms have been characterized in cancer cells, and there is no description of them in other tissues. Additionally, a recent study (Krishnan et al., 2022) reported the inability to detect p75 CUX1 in cancer cells, further emphasizing the need for careful interpretation of CUX1 isoform functions in diverse tissues.

Remarkably, a number of investigations have shed light on the additional roles of certain TFs as accessory factors that enhance the enzymatic activities of DNA repair proteins. Although CUT domains were initially identified based on their DNA-binding capabilities (Andrés et al., 1994; Aufiero et al., 1994; Harada et al., 1994), more recent studies have demonstrated that the CUT domains found in various CUT homeobox proteins (including CUX1, CUX2 and SATB1) are also implicated in DNA repair through protein–protein interactions (Ramdzan et al., 2014; Kaur et al., 2016). These investigations have provided insights into the functional role of CUT domains as accessory proteins that enhance DNA repair processes by promoting the enzymatic activities of key base excision repair (BER) enzymes, including APE1, OGG1, and DNA polβ (Ramdzan et al., 2021b). BER is a DNA repair pathway that plays a critical role in repairing the majority of oxidative DNA damage lesions (Dianov and Hübscher, 2013). The downregulation of *Cux1* has been shown to hinder the DNA repair process when cells are exposed to hydrogen peroxide or ionizing radiation (Kaur et al., 2018). Conversely, the overexpression of *Cux1* boosts DNA repair (Ramdzan et al., 2014). The involvement of CUX proteins in oxidative repair processes suggests a potential connection between *Cux* genes and the maintenance of neuronal health. Given the elevated metabolic activity in neurons, which results in the generation of substantial amounts of reactive oxygen species (ROS), effective repair of oxidative DNA damage becomes essential to uphold the integrity of the neuronal genome and maintain overall neuronal well-being (McKinnon, 2013). In the comprehensive review of *Cux* genes by Weiss and Nieto (2019) it is hypothesized that the duplication of the CUX gene during evolution might have been advantageous due to the protective role exerted by CUX proteins against oxidative DNA damage in the nervous system. Notably, when *Cux2* was downregulated in cortical neurons, it resulted in an elevated level of oxidative DNA damage (Pal et al., 2015). Defects in BER have been detected both in the nervous system of aging rats and in individuals affected by mild cognitive impairment and Alzheimer’s disease (Weissman et al., 2007). These findings suggest that the roles played by CUX proteins may serve as protective mechanisms against neurodegeneration, contributing to the longevity of neurons.

## 9. CUT homeobox genes in human disease

In vertebrates, CUT homeobox genes have emerged as key regulators of tissue development, and their deregulation has been linked to human diseases. Notably, in the context of cancer, the expression and functions of SATB1/2 and CUX1 have been extensively investigated in numerous studies (Hulea and Nepveu, 2012; Ramdzan and Nepveu, 2014; Naik and Galande, 2019). Moreover, differentiation defects in any of the multiple cell types where CUT homeobox genes are expressed could predispose an individual to developmental diseases.

The cellular functions of CUX1 reviewed above indicate potential mechanisms through which increased expression of p110 or p75 isoforms may contribute to tumor development and progression. These mechanisms include the facilitation of S phase entry, promotion of cell motility and invasion, as well as conferring resistance to apoptosis [reviewed in Hulea and Nepveu (2012)]. The initial indication of CUX1 role in cancer arose from observations of its overexpression in primary tumors and cancer cell lines. Subsequent studies in mouse further validated its role in cancer. Transgenic mice engineered to express p200 CUX1 under the control of the cytomegalovirus (CMV) promoter developed multiorgan organomegaly, which aligns with a model wherein CUX1 promotes the proliferation of progenitor cells while not impeding terminal differentiation processes (Ledford et al., 2002). A second transgenic mouse, overexpressing the p75 human CUX1 isoform developed tumors (Cadieux et al., 2006).

Therefore, the evidence indicates that increased CUX1 can promote tumor progression (*CUX1* as an oncogene). However, in contradiction, additional evidence supports a model of haploinsufficiency, where reduced CUX1 expression contributes to tumor development (*CUX1* as a tumor suppressor gene). Indeed, CUX1 inactivating mutations and loss of heterozygosity have been identified as contributing factors in various human cancers (Ramdzan and Nepveu, 2014; Wong et al., 2014). Given the dual roles of CUX1 as both an oncogene and a tumor suppressor depending on the context, CUX1 molecular roles have been extensively studied to explore its potential associations with DNA repair processes. Comprehensively reviewed by Ramdzan et al. (2021a), cancer cells take advantage of the functions of CUT proteins in DNA repair to promote their survival. In particular, cancer cells with an activated RAS pathway generate excessive amounts of ROS. In the absence of an adequate increase in antioxidant production, ROS accumulation leads to persistent oxidative DNA damage and eventual cell death. In response to increased ROS levels, cancer cells upregulate CUT genes to enhance their ability to repair oxidative DNA damage. Interestingly, an increase on CUX1 expression was found to cooperate with RAS in promoting tumor formation in mice (Ramdzan et al., 2014). In contrast, downregulation of CUT genes was found to be lethal in cancer cells with elevated ROS levels. Notably, this adaptation confers increased resistance to genotoxic treatments in cancer cells that overexpress CUT proteins. Furthermore, CUX1 was associated with the regulation of proteasome-mediated degradation, which can impact the migratory and invasive properties of tumors (Aleksic et al., 2007).

Moreover, in recent studies, *CUX2* variants were found to be associated with a rare developmental and epileptic encephalopathy, with onset in infancy and characterized by refractory seizures, global developmental delay, movement disorders, speech delay, and autistic behavior (Barington et al., 2018; Chatron et al., 2018; Zhang et al., 2022). Another study found multiple *CUX2* and *CASP* (CUX1 alternatively spliced product, an alternative *CUX1* isoform) variants in patients with temporal lobe epilepsy. Results in knockout mice showed that these variants affect neuronal properties and enhance excitatory synaptic transmission (Suzuki et al., 2022).

The SATB family of chromatin organizers plays distinct roles in cancer progression, specifically in the regulation of cell proliferation and migration, apoptosis, metastasis, and angiogenesis [reviewed in Kohwi-Shigematsu et al. (2013) and Naik and Galande (2019)]. SATB proteins are involved in orchestrating the complex interplay between these processes, thereby influencing the overall course of cancer development and progression. SATB1 has been shown to have an impact on chromatin structure and interacts with various transcriptional cofactors during tumor development. Conversely, SATB2 exhibits differential expression in various cancer types and plays a role in tumor formation. One specific example is the identification of SATB1 as a contributing factor to the development of aggressive phenotypes in breast cancer (Han et al., 2008).

ONECUT1 has been implicated in cancer due to its involvement in preventing epithelial-to-mesenchymal transition in cancer cells through its interaction with p53. This activity highlights the potential tumor suppressive role of ONECUT1 (Yuan et al., 2013). Moreover, ONECUT factors have been associated with liver and pancreas disease. Liver ductal plate malformations are attributed to defects in biliary tract development and contribute to both Jeune Syndrome and Meckel Syndrome in humans. Inactivation of *Onecut1* leads to ductal plate malformations including hepatic artery malformations (Clotman et al., 2003). *Onecut1* is also involved in liver cancer by regulating miR-122, a tumor suppressor microRNA (Nakao et al., 2014). This microRNA plays a crucial role in preventing liver cancer by repressing the expression of tumorigenic genes. The involvement of ONECUT1 in the regulation of miR-122 underscores its significance in liver cancer and highlights its potential as a therapeutic target for this disease.

In the nervous system, *CUX1* has been linked with Autism Spectrum Disorder (ASD), and with impaired intellectual development (Doan et al., 2016; Liu et al., 2016; Platzer et al., 2018). On the other hand, mutations in *CUX2* have been linked to bipolar disorder, seizures and ASD (Glaser et al., 2005; Barington et al., 2018). There is a potential connection between brain disorders and the functions of CUT genes during the development of the corpus callosum. As reviewed above, *Cux1* plays a role in activity-dependent mechanisms contributing to circuit assembly. In mice, animals lacking *Cux1* display aberrant firing responses and selective loss of corpus callosum contralateral innervation (Rodríguez-Tornos et al., 2016). Abnormal formation of the corpus callosum in humans is linked with various neurological conditions that exhibit a wide array of symptoms, such as attention deficits, language dysfunction, impaired social interaction, and diminished self-awareness (David et al., 1993; Paul et al., 2007; Lau et al., 2013; Sarrazin et al., 2014, 2015). SATB2 was initially discovered as a gene associated with disease for the cleft palate, among additional craniofacial alterations (FitzPatrick et al., 2003; Britanova et al., 2006; Rosenfeld et al., 2009; Balasubramanian et al., 2011). Mutations in *SATB2* lead to “SATB2-associated syndrome,” a complex disorder affecting multiple systems marked by severe neurodevelopmental impairments, including behavioral complications and speech difficulties (Dobreva et al., 2006; Leoyklang et al., 2007; Alcamo et al., 2008; Britanova et al., 2008). In addition, SATB2 is associated with multiple neurodevelopmental diseases including schizophrenia (Liedén et al., 2014; Lee et al., 2016; Whitton et al., 2016). As noted by Kropp and Gannon (Kropp and Gannon, 2016), although no direct links have been established between ONECUT factors and neuronal diseases, it is plausible that impaired differentiation in various neuron types could contribute to disease development. For instance, the regulation of mesodiencephalic dopaminergic neurons by ONECUT1 is particularly relevant as these neurons are connected to the development of Parkinson’s disease (Smidt and Burbach, 2007; Chakrabarty et al., 2012).

## 10. Evolutionary dynamics of the CUT homeobox gene class

Excellent works on the evolution of CUT homeobox genes have been previously published (Bürglin and Cassata, 2002; Takatori and Saiga, 2008). Here I highlight a specific aspect of the distinctive and intricate evolution of this gene class, indicating a remarkable evolutionary trajectory in the lineage to vertebrates. The structural organization of the CUX genes is highly unique, as they share the N-terminus with a distinct gene named CASP. Interestingly, CASP is present as a separate gene in yeast and plants (Bürglin and Cassata, 2002). CASP is known to localize to the Golgi membrane, and is predicted to form a coiled-coil structure at the N-terminal region. This structural feature could potentially play a role in protein–protein interactions in CUX proteins (Gillingham et al., 2002). During the course of evolution, there was a significant event where an ancestral CUX gene became interconnected with the CASP gene by alternative splicing. CASP shares the N-terminal region with CUX but is lacking the CUT domains and the homeodomain. This association is observed from nematodes to vertebrates, except in *Drosophila* where the CASP gene is absent (Lievens et al., 1997). Remarkably, there are differences in the organization of the CUX/CASP gene between *C. elegans* and vertebrates. In mouse, the CASP specific exons are located in between CUX specific exons, whereas in *C. elegans*, the CASP specific exons are downstream of the exons encoding the TF. The analysis of the genome of the amphioxus *Branchiostoma floridae* shed light into the evolution of this locus. The identification of a nematode like *CUX/CASP* structure in the genome of *B. floridae* suggests that the significant evolutionary change in the genomic structure of the locus possibly happened by domain shuffling within the gene after the divergence of amphioxus and vertebrate ancestors (Takatori and Saiga, 2008). Strikingly, in the human *CUX1* gene the CASP specific exons are again found downstream of the exons encoding the DNA binding domains. Another key difference between human and mice lies in the genomic location of *Cux* genes. While they are located in the same chromosome in mice (*Mus musculus*, chromosome 5) and rats (*Rattus novergicus*, chromosome 12); in humans, as well as in other vertebrates such as zebrafish, they are located in different chromosomes. The fusion of two genes via alternative splicing represents a rare phenomenon that is not observed in any other homeobox gene.

## 11. Conclusion

In summary, CUT homeobox genes exhibit widespread expression in multiple tissues and play critical roles in promoting differentiation and maturation of diverse cell types. While their significant expression during development is well established, the persistent expression of CUT genes suggests their involvement in maintaining the mature differentiated state of multiple cell types, potentially conferring protective effects against disease. Notably, CUT factors employ distinct mechanisms of gene expression regulation, engaging in both classic and non-canonical interactions with DNA, an area that continues to be actively researched. The significance of CUT genes extends beyond developmental processes into disease pathogenesis, as they hold promise as therapeutic targets or prognostic factors in cancer. Furthermore, their involvement in cell fate specification offers exciting opportunities for manipulating cell differentiation in tissue repair. Additionally, their role in DNA repair mechanisms presents prospective implications for the treatment of human diseases, particularly cancer. The integration of genetic studies and genomic technology will be indispensable to further advance our understanding of CUT homeobox gene functions during development and disease. Exploring the intricate regulatory networks and deciphering the precise roles of CUT homeobox genes will undoubtedly deepen our knowledge of these fascinating genes and open new avenues for innovative therapeutic approaches.

## Author contributions

EL-D: conceptualization, writing – original draft, and writing – review and editing.

## Funding

EL-D was funded by a CIDEGENT grant from Generalitat Valenciana (CIDEXG/2022/30), and by a Marie Skłodowska-Curie Action from European Union (101064583-CELLIDRECUT-HORIZON-MSCA-2021-PF-01).

## Conflict of interest

The author declares that the research was conducted in the absence of any commercial or financial relationships that could be construed as a potential conflict of interest.

## Publisher’s note

All claims expressed in this article are solely those of the authors and do not necessarily represent those of their affiliated organizations, or those of the publisher, the editors and the reviewers. Any product that may be evaluated in this article, or claim that may be made by its manufacturer, is not guaranteed or endorsed by the publisher.
